# A Tailored, Multi-Component Falls Prevention Intervention for Community-Dwelling Older Adults in Singapore

**DOI:** 10.1093/geroni/igaf122.982

**Published:** 2025-12-31

**Authors:** Jing Wen Goh, Vanessa Koh, Kok Yang Tan, Angelique Chan, Navrag Singh, David Matchar

**Affiliations:** Duke-NUS Medical School, Singapore, Singapore; Duke-NUS Medical School, Singapore, Singapore; Duke-NUS Medical School, Singapore, Singapore; Duke-NUS Medical School, Singapore, Singapore; Institute for Biomechanics ETH Zürich, Zürich, Zurich, Switzerland; Duke-NUS Medical School Programme in Health Services & Systems Research, Singapore, Singapore

## Abstract

Despite strong evidence supporting personalized, multi-component falls prevention programs, many existing programs in Singapore focus primarily on exercise and lack individualization. This study aims to address this gap by assessing the effectiveness of a tailored multi-component program, integrating both exercise and education, to reduce falls and injurious falls among community-dwelling older adults at high risk for falls. A 12-month, randomized controlled trial was conducted, where the intervention group participated in a tailored exercise and education program twice a week for 12 weeks, while the control group received falls prevention booklet. Intention-to-treat (ITT) and per protocol analyses (PPA) were conducted to assess the impact on number of fallers and injurious fallers, physical performance (assessed using the Short Physical Performance Battery, SPPB), falls efficacy (assessed using the Falls Efficacy Scale, FES), and falls prevention behaviors (assessed using the Falls Behavioral Scale, FaB). Preliminary results from both ITT and PPA analyses demonstrated that the intervention group had positive improvements in the SPPB lower limb strength subscale compared to the control group, along with improvements in falls efficacy and falls prevention behaviors. The PPA analysis also revealed a positive improvement in the SPPB total score and balance subscale for the intervention group. These preliminary findings highlight the need for a multi-component, tailored falls prevention program, as such a program not only improves physical performance, but also increases awareness and knowledge about falls and falls prevention; resulting in positive behavioral changes and more proactive approach to safety.

